# Editorial: Emerging talents in human neuroscience: cognitive neuroscience 2023

**DOI:** 10.3389/fnhum.2024.1488829

**Published:** 2024-09-18

**Authors:** Peter M. Vishton, Sébastien Hélie

**Affiliations:** ^1^Department of Psychological Sciences, William & Mary, Williamsburg, VA, United States; ^2^Department of Psychological Sciences, Purdue University, West Lafayette, IN, United States

**Keywords:** cognitive neuroscience, intrinsic motivation, cognitive effort, EEG, decision making, cognitive flexibility, attention, short video use

We are pleased to present the *Emerging talents in human neuroscience: cognitive neuroscience 2023* article Research Topic. The Research Topic highlights meaningful contributions made by emerging experts in this important domain of research. It has been our firm belief, from the inception of this topic, that junior–even student–researchers often conduct high-quality studies that lead to important contributions to our understanding of how the human brain implements cognitive processes.

Indeed, it is often someone who is “new” to a research area who is most likely to ask questions that shake the foundations of that area. “I understand why you might think that, Dr. Professor, but couldn't you also explain the result in this other way?” Our own research groups have made some of our most significant advances on the shoulders of such questions. The words of young researchers are highly worthy of consideration.

Much of the work from relatively junior researchers does not reach a wide, international audience, however, due to the daunting process of shepherding research findings through the gauntlet of peer review to publication. This Research Topic specifically sought out projects that were led by student researchers, from the conception of the fundamental idea of the project through to the design, implementation, and reporting of the study. Four excellent examples of this make up the Research Topic presented here. True to form for young investigators, all four articles do much more than reify existing theories of cognitive function. Indeed, all four call for a reconsideration and/or reframing of our approach to a fundamental topic in cognitive neuroscience.

Schneider et al. explored the cognitive mechanisms involved in recognition memory, particularly how the brain distinguishes between different types of memory-related decisions. The research challenges the dual-process model, which traditionally separates recognition into two distinct processes: familiarity (a fast, automatic recognition process) and recollection (a slower, more deliberate process). The study argues that recognition memory can only be fully understood as emerging from an interaction of bottom-up processes (e.g., memory strength) and top-down processes (e.g., decision-making strategies).

The study's methodology involved a challenging visual recognition task, in which participants were shown a series of images and asked to identify whether they had seen them previously. Four possible “signal detection” outcomes are possible across trials: (1) correct identification of a familiar image, (2) correct identification of a novel image, (3) incorrect belief that an unfamiliar image was familiar, and (4) incorrect belief that a previously seen image was novel. Event-related potential (ERP) data were collected during this task. The researchers identified a significant time window (470 to 670 ms post-stimulus) during which the brain's electrophysiological activity differed across the four responses. Notably, the study found that the posterior-left cluster of electrodes distinguished all four outcomes, while the fronto-central cluster only differentiated responses based on whether participants responded “yes” or “no.”

These findings suggest that recognition memory processes might be better explained by considering both bottom-up factors, like memory strength, and top-down factors, like decision-making processes, rather than strictly separating familiarity and recollection as the dual-process model does. This study contributes to the ongoing debate by emphasizing the role of decisional factors in recognition memory and demonstrating the value of using a comprehensive, model-free approach to analyze ERP data.

Hohl and Dolcos provide a comprehensive overview of cognitive flexibility (CF), a crucial aspect of executive function that enables individuals to adapt their thinking and behavior in response to changing circumstances. The authors highlight that while CF is widely acknowledged as beneficial, its conceptualization and measurement remain inconsistent across different fields.

The review begins by discussing the varied definitions of CF. It is often used interchangeably with terms like psychological flexibility, mental flexibility, and behavioral flexibility, leading to confusion in its operationalization. The authors identify four primary conceptualizations of CF: as an ability or skill, a property of cognitive states, a personality trait, and an outcome of divergent thinking or creativity. Despite these varied definitions, the most common view is that CF is a core aspect of executive function, specifically involving the ability to shift between tasks or mental sets.

The article then explores three main approaches to assessing CF: neuropsychological tasks, self-report questionnaires, and neuroscientific methods. Neuropsychological tasks, such as task-switching paradigms and the Wisconsin Card Sorting Test, are designed to measure shifts in cognition or behavior in response to changing environmental demands. However, these tasks often suffer from the “task-impurity problem,” where the assessment might engage other executive functions beyond CF. Self-report questionnaires, such as the Cognitive Flexibility Inventory, measure an individual's perceived ability to adapt their thinking and behavior in everyday life but are susceptible to biases like social desirability. Neuroscientific approaches, including functional MRI and EEG, identify the brain regions and networks involved in CF, such as the frontoparietal network, providing insights into the neural underpinnings of cognitive flexibility.

The review calls for a more integrative approach to understanding and measuring CF, combining neuropsychological, self-report, and neuroscientific methods. The authors argue that such a multimethod approach, grounded in the interactions between behavior, brain, and context, is essential for advancing our understanding of CF and developing effective interventions to enhance it. This comprehensive framework could lead to more consistent definitions, better assessment tools, and more effective interventions for improving cognitive flexibility in various domains of life.

Yan et al. explored the effects of excessive short-form video consumption on attentional functions. With the rapid rise of platforms like TikTok and Instagram Reels, there is growing concern about their potential negative impact on users' mental health, particularly on cognitive functions like attention. The researchers used the Attention Network Test (ANT) and electroencephalogram (EEG) recordings to examine how these addiction tendencies affect executive control, a key component of attention. The study found a significant negative correlation between addiction tendencies and the theta power index in the prefrontal cortex (r = −0.395, *p* = 0.007). This suggests that higher addiction tendencies are associated with reduced executive control, indicating impaired attentional functions. These findings suggest that excessive short-form video use could detrimentally affect cognitive functions, particularly those related to attention and self-control.

The observed decrease in theta power among participants with higher addiction tendencies highlights the potential neural mechanisms underlying these cognitive deficits–and perhaps addiction in other contexts as well. The research also provides a foundation for future studies to explore interventions aimed at reducing the adverse effects of excessive short-form video consumption.

Randez and Hélie investigated how levels of cognitive effort influence human decision making processes. The study focuses on two key components: intrinsic motivation and capability. Intrinsic motivation refers to the drive to perform a task for its own sake, while capability relates to an individual's ability to complete the task. The authors argue that previous research has often confounded these two factors, leading to unclear interpretations of what motivates people to engage in effortful tasks. An adaptive algorithm was used to adjust the difficulty of tasks based on each participant's performance. Participants were then asked to choose between tasks with varying levels of demand, with the study aiming to reveal whether preferences for high- or low-demand tasks were driven by intrinsic motivation or capability.

Intrinsic motivation levels played a clear and significant role on decision-making, independent of levels of capability. While some individuals were motivated to take on more challenging tasks, others preferred lower-demand options, even when the task-demands were equal. This suggests that individual differences in intrinsic motivation can lead to different patterns of effort-based decision-making, which has important implications for understanding how people engage in tasks requiring cognitive effort. The studies are interesting in their own right, but the methods developed open the door to a wide range of additional questions about what drives preference for different levels of task demand. Have the Randez and Hélie identified a new personality characteristic? How do fatigue and risk tolerance interact with this demand preference?

All four articles describe a deep engagement within a classic area of cognitive neuroscience. All four call for a reformulation of the fundamental theories that have described the phenomena in these areas for decades. Are they correct? We cannot know at present. But correct or not, determining the validity of the assertions offered in these articles will certainly lead to a better understanding of human cognitive processes.

